# Supplementary material for: MetaRibo-Seq measures translation in microbiomes
Source: Nat Commun. 2020 Jun 29;11:3268. doi: 10.1038/s41467-020-17081-z (PMC7324362; doi:10.1038/s41467-020-17081-z)
Supplement: Supplementary file 10 — Supplementary Data 7 [file 41467_2020_17081_MOESM10_ESM.zip › File2/Confidence_High_Taxonomy/260075_out.krona.html]

Javascript must be enabled to view this page.

members
magnitude
magnitudeUnassigned
count
unassigned
taxon
rank

260075\_out

67

2
superkingdom
67

phylum
66
1239

186801
class
66

186802
order
66

family
1
541000

216851
genus

SRS104084\_contig\_number\_3347
1

7
family
186803

1898203

SRS147919\_contig\_number\_7013
species
1

1

SRS142542\_contig\_number\_14395
species
658655

572511
genus
5

2292967
5

SRS012969\_contig\_number\_contig-100\_2970.177468SRS014683\_contig\_number\_13726SRS042628\_contig\_number\_7997SRS143417\_contig\_number\_contig-100\_2959.140045SRS144135\_contig\_number\_contig-100\_2540.207284
species

58
family
31979

genus
57
1485

2
species

SRS103987\_contig\_number\_37336SRS149784\_contig\_number\_27688
2292206

2293041
species

SRS011134\_contig\_number\_contig-100\_2488.264884SRS011239\_contig\_number\_26716SRS013800\_contig\_number\_15436SRS015431\_contig\_number\_16101SRS016018\_contig\_number\_contig-100\_1105.87133SRS016056\_contig\_number\_7221SRS017247\_contig\_number\_contig-100\_231.69397SRS017307\_contig\_number\_contig-100\_941.172237SRS017916\_contig\_number\_18362SRS018427\_contig\_number\_12128SRS018817\_contig\_number\_contig-100\_1108.100743SRS019178\_contig\_number\_contig-100\_1548.36268SRS019445\_contig\_number\_9462SRS020233\_contig\_number\_35523SRS021484\_contig\_number\_28044SRS024075\_contig\_number\_contig-100\_1283.99222SRS024435\_contig\_number\_contig-100\_1288.240954SRS045195\_contig\_number\_contig-100\_858.63355SRS047433\_contig\_number\_contig-100\_712.127994SRS049712\_contig\_number\_7726SRS049773\_contig\_number\_contig-100\_1489.203815SRS050752\_contig\_number\_contig-100\_3087.135558SRS052697\_contig\_number\_contig-100\_886.246780SRS053398\_contig\_number\_23924SRS062654\_contig\_number\_contig-100\_2361.121379SRS063370\_contig\_number\_contig-100\_451.88316SRS063813\_contig\_number\_4865SRS063985\_contig\_number\_contig-100\_1433.168176SRS065176\_contig\_number\_16226SRS075821\_contig\_number\_21976SRS077294\_contig\_number\_16229SRS077730\_contig\_number\_18323SRS077849\_contig\_number\_contig-100\_4477.159324SRS098073\_contig\_number\_17732SRS098571\_contig\_number\_63554SRS1041038\_contig\_number\_11645SRS104485\_contig\_number\_contig-100\_1791.28986SRS104636\_contig\_number\_16235SRS1055076\_contig\_number\_16997SRS142503\_contig\_number\_2722SRS143466\_contig\_number\_29218SRS143876\_contig\_number\_contig-100\_1429.233577SRS144506\_contig\_number\_56641SRS147139\_contig\_number\_16139SRS147377\_contig\_number\_contig-100\_1563.78010SRS147614\_contig\_number\_contig-100\_2081.35546SRS148091\_contig\_number\_5780SRS148817\_contig\_number\_14112SRS148874\_contig\_number\_contig-100\_2315.90098SRS149325\_contig\_number\_contig-100\_1867.91454SRS893252\_contig\_number\_contig-100\_1255.118908SRS893292\_contig\_number\_3305SRS893295\_contig\_number\_12228SRS893300\_contig\_number\_508SRS971276\_contig\_number\_36617
55

420345
genus
1

1
species

SRS098881\_contig\_number\_5247
341220

976
phylum
1

200643
class
1

order
1
171549

1853231
family
1

283168
1
genus

2292236
species

SRS023526\_contig\_number\_34714
1
